# Bioavailability of Tea Catechins and Its Improvement

**DOI:** 10.3390/molecules23092346

**Published:** 2018-09-13

**Authors:** Zhuo-Yu Cai, Xu-Min Li, Jin-Pei Liang, Li-Ping Xiang, Kai-Rong Wang, Yun-Long Shi, Rui Yang, Meng Shi, Jian-Hui Ye, Jian-Liang Lu, Xin-Qiang Zheng, Yue-Rong Liang

**Affiliations:** 1 Tea Research Institute, Zhejiang University, Hangzhou 310058, China; 21716160@zju.edu.cn (Z.-Y.C.); 21616096@zju.edu.cn (X.-M.L.); 11516051@zju.edu.cn (Y.-L.S.); 21616106@zju.edu.cn (R.Y.); 11616052@zju.edu.cn (M.S.); jianhuiye@zju.edu.cn (J.-H.Y.); jllu@zju.edu.cn (J.-L.L.); 2 Intellectual Property Office of Lanshan District, Rizhao 543003, China; liangjinpeirz@163.com; 3 National Tea and Tea Product Quality Supervision and Inspection Center (Guizhou), Zunyi 563100, China; xlping6009@126.com; 4 Ningbo Extension Station of Forestry & Speciality Technology, Ningbo 315012, China; wkrtea321hjytea@163.com

**Keywords:** *Camellia sinensis*, catechins, bioavailability, nanoparticle, interaction

## Abstract

Many in vitro studies have shown that tea catechins had vevarious health beneficial effects. However, inconsistent results between in vitro and in vivo studies or between laboratory tests and epidemical studies are observed. Low bioavailability of tea catechins was an important factor leading to these inconsistencies. Research advances in bioavailability studies involving absorption and metabolic biotransformation of tea catechins were reviewed in the present paper. Related techniques for improving their bioavailability such as nanostructure-based drug delivery system, molecular modification, and co-administration of catechins with other bioactives were also discussed.

## 1. Introduction

Increased evidence has expanded the role of green tea from a traditional beverage to a source of bioactive ingredients with many health benefits. Green tea is rich in catechins comprised of more than eight polyphenolic compounds. According to different sources, the most abundant catechins in tea are (−)-epigallocatechin gallate (EGCG) and (−)-epigallocatechin (EGC) [1,2]. In recent years, EGCG has attracted significant research interest due to its beneficial health effects including antioxidation [3], anti-diabetes [4], anti-inflammation [5,6,7] and anti-tumorigenesis activity [8]. Therefore, EGCG has recently gained the attention of scientists for implementation as a therapeutic alternative for treating some diseases.

There have been many in vitro studies showing that EGCG possesses antioxidant properties and exhibits favorable effects on gene expression, signal transduction and other cell functions [9]. Numerous potential mechanisms have been proposed to explain the healthy benefits of tea catechins, including improving antioxidative activity, suppressing adipocyte differentiation, regulating the tumor-suppressor microRNAs, inhibiting hepatocyte growth factor receptor activity, inhibiting Iκb kinase activity, etc. [3,10,11,12,13]. In vitro studies showed that the effective concentrations of EGCG ranged from 1 to 100 μmol/L. However, the peakplasma levels of tea catechins were usually in the sub-or low-micromolarrange in human subjects or animals following oraladministration of green tea catechins [14,15], which was lower than the effective concentration of in vitro tests. Therefore, the therapeutic effect is limited, owing to poor stability in the gastrointestinal tract and limited membrane permeability across the intestine [16,17,18]. Their therapeutic potential is limited by its poor systemic absorption following oral administration, including low absorption, poor pharmacokinetics and bioavailability, scarce biodistribution, first-pass metabolism, trivial penetration and low accumulation in the related tissues of the body, or low targeting efficacy. The inconsistency between catechins’ superior in vitro biological activity and low absorption in in vivo studies can also be attributed to its low stability, which lead to the formation of degradation products and pro-oxidant molecules [19]. Catechins are unstable under physiologic conditions and they could be rapidly degraded or metabolized through interactions with the hydroxyl groups on the phenol rings [19]. Even if administered intravenously, catechins were partially degraded before reaching the target tissues [20]. EGCG needs to work at a relatively high concentration to target related molecules and to affect disease-related cellular processes [9,21]. It is believed that taking green tea polyphenol products in amounts equivalent to the EGCG content in 8–16 cups of green tea daily may mitigate the poor bioavailability of EGCG [22]. The systemic availability of EGCG increased at higher doses, possibly due to saturable pre-systemic elimination of orally administered green tea polyphenols (GTPs) [23]. However, it is obviously not a good idea to use excessive dose of catechins for improving effectiveness. Studies from experimental animals and epidemiological surveys have shown that GTPs have a dose-dependent toxicology [24]. Tea polyphenols are antioxidants, but they can also generate reactive oxygen species (ROS). It was observed that moderate doses of tea polyphenols induced finite amount of lower level of ROS which may also activates nuclear factor erythroid 2-related factor 2 (Nrf2) to activate antioxidant and detoxifying enzymes, and then attenuate oxidative stress, whilst excessive amounts of GTPs probably induced a pro-oxidant effect, resulting in induced toxicity effect [9].

The present paper comprehensively reviews the mechanisms leading to low absorption, poor permeability and less stability of tea catechins and also describes the potential for improving the bioavailability of tea catechins through new techniques such as nanoparticle-based delivery systems, structurally modified molecule of catechins, co-administration with other drugs or bioactives. Furthermore, the challenges and future research directions are also discussed.

To collect the related references, computerized systematic literature searches were conducted inWeb of Science and Google Scholar databases to retrieve the pertinent studies and reviews. The search keywords used were tea catechins or tea polyphenolsor epigallocatechin gallate (EGCG) in combination with the terms absorption, bioavailability, metabolism, or nanoparticles, chitosan, prodrugs, peracetylate, and synergistic as well as the terms improvementand enhancement. The papers published in the English languagewere exclusively evaluated. No other limitations were applied. The literatures were combed and divided into four main categories: (a) pharmacokinetics of tea catechins, (b) drug delivery system, (c) molecular modification, and (d) synergistic effect with other drugs. Thus, the review is structured. First, into a discussion on the bioavailability of catechins, and then, to provide more thorough insights into the way and mechanism to improve.

## 2. Absorption and Metabolism of Tea Catechins

Only a small fraction of tea catechins present in the intestinal tract after drinking tea can be absorbed, and therefore considered to be bioavailable, i.e., appearing in the blood and tissues or reaching the systemic circulation. The latest study demonstrates that concentration of catechins in green tea infusions is about 3250–4410 mg/L [2]. Tests showed that less than 5% of the orally administered dose of tea catechins reached the systemic circulation in rat [25,26] and approximately 1.68% of ingested catechins were present in human’s plasma (0.16%), urine (1.1%) and feces (0.42%) after tea ingestion over 6 h [27]. The pharmacokinetics study showed that after oral administration of tea to rats, about 14% of (−)-epigallocatechin (EGC), 31% of (−)-epicatechin (EC), and <1% of (−)-epigallocatechin-3-gallate (EGCG) appeared in the blood [28]. For humans, after administration of 3 g of decaffeinated green tea, the maximum plasma concentration for EGCG, EGC, and EC were 0.57, 1.60, and 0.6 μM, respectively [29].

A fraction of the ingested tea catechins undergoes extensive metabolism by phase II enzymes, such as UDP-glucuronosyltransferases (UGTs), sulphotransferases (SULTs) and catechol-*O*-methyltransferase (COMT) before and after being absorbed predominantly in the small intestine and in liver [30], with the remained catechins entering the colon. Partial catechins pass into colon with the secreted bile in an enterohepatic recirculation process, where they are degraded into different flavonoid rings by the resident microorganisms. The metabolites identified in humans include glucuronide and sulfate conjugates, methylated tea catechin conjugates, and microflora-mediated ring fission products and phenolic acid catabolites [31]. The catabolized phenolic acids can be re-absorbed into the circulation or excreted into urine. Stalmach et al. [32,33] reported that when human subjects with an ileostomy consumed green tea solution containing 634 μmol of catechins, 70% of the ingested catechins was detected in the ileal fluid during 0–24 h after administration, in which 33% was in the form of parent compounds and 37% was the metabolites with 23 compounds in total. Sixteen of the metabolites were found in plasma, in the forms of principally methylated, sulphated and glucuronide conjugates of catechins, with peak catechins concentration 101–256 nM in plasma. Catechins and their conjugated metabolites, catabolized small molecular phenolic acids can be distributed in various organs and tissues, where they perform various biological actions.

No specific receptors on the surface of small intestinal epithelial cells have been found to carry EGCG into cells. Thus, the mechanism for catechins being transported across epithelium is principally based on passive diffusion, including paracellular and transcellular diffusions. Cell studies have also suggested that EGCG underwent active efflux after absorption by ATP-depended proteins. EGCG and its metabolites were served as common substrates for two important protein efflux pumps including the multidrug resistance-associated protein (MRP) efflux pumps [34] and the P-glycoprotein (P-gp) [35,36], by which majority of the absorbed catechins were pumped back to the extracellular or intestinal space, resulting in limitation of their bioavailability.

**Figure 1:**
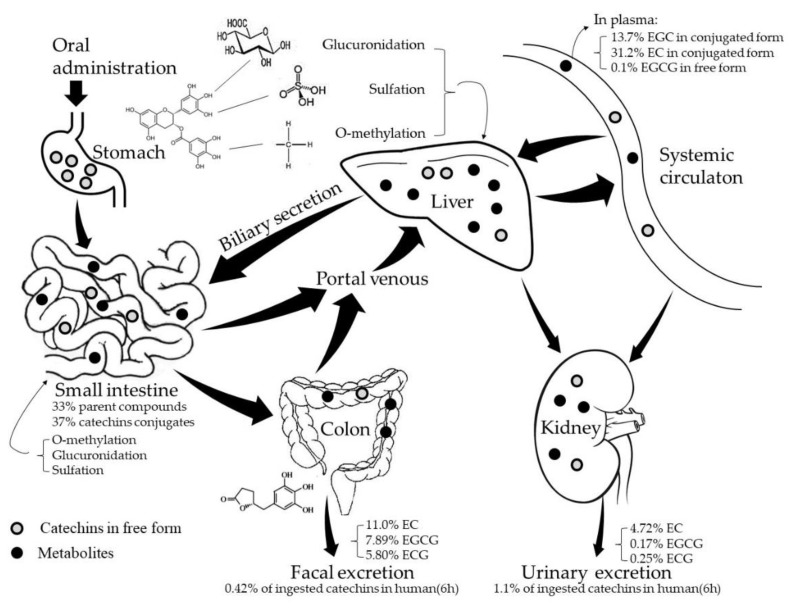
Schematic diagram of metabolism of green tea catechins [28,32,33,37].

In brief, many processes including chemical degradation, microbial metabolism, intestinal and hepatic metabolism, membrane permeability, and transporter-mediators may influence the bioavailability of catechins (Figure 1).

## 3. Improving the Bioavailability of Catechins

### 3.1. Nanostructure-Based Drug Delivery System

Nanostructure-based drug delivery system is one of the fastest-emerging areas in improving the bioavailability of drugs. Many studies showed promising EGCG-loaded nano-carriers with sustained release and improved bioavailability even at much lower doses than conventional preparations. Encapsulation materials including lipids [38,39], proteins [40,41], carbohydrate [42] can be used as carriers and exert improving effects on the bioavailability of diet catechins including EGCG, via enhancing its solubility, preventing its degradation in the intestinal environment, elevating the permeation in small intestine, resulting in an increased concentration in the bloodstream [43,44].

#### 3.1.1. Protein-Based Carriers

The stability of EGCG was improved when bound to bovine serum albumin (BSA) [45]. Both EGCG ovalbumin−dextran conjugate nanoparticle and chitosan coated BSA–EGCG nanoparticle showed significantly higher apparent permeability coefficient compared to free EGCG in solution, resulting in an improvement of the EGCG absorption [46,47]. The radioprotection effect of chitosan coated BSA–polyphenols nanoparticles by oral administration in mice was significantly higher than that of free polyphenols [48].

A catechin-loaded nanoemulsion-based nanogel showed sustained release profile, resulting in enhanced photoprotection potential to skin due to its improved permeability as well as bioavailability through transdermal route, compared to the conventional gel [49], in which gelatin showed higher bioaccessibility and antioxidant activity than chitosan [50].

Milk proteins, containing caseins and whey proteins, were considered as ideal carriers for delivering catechins. Sodium caseinate adsorbed at the oil water interface, can load high ratios of EGCG [51,52]. Casein micelles can be used as protective carriers for EGCG in foods. It was demonstrated that nanoencapsulation of EGCG in casein micelles did not diminish antiproliferative activity of the catechins on colon cancer cells, compared with free form EGCG [53]. Accordingly, casein micelle is considered to be an ideal platform for catechin delivery while the binding of caseins with EGCG would not affect the bioaccessibility of EGCG.

Other proteins such as corn zein protein [41,54], soy protein [55] and rice bran protein isolate [56] were confirmed to improve the stability, bioaccessibility and permeability of catechins, and could be employed as satisfactory carriers for tea catachins. Incorporation of EGCG in zein fiber-forming solution through hydrogen bonding, hydrophobic interactions, and physical encapsulation enhanced the stabilization of EGCG [41].

The presence of hydroxyl groups in EGCG contributes to its strong affinity to proteins by enhancing its interactions with proteins. Although the interactions between catechins and proteins alter the protein structure [57], leading to partial decrease in antioxidant activity of EGCG, the protein protects EGCG against degradation [58].

#### 3.1.2. Carbohydrate-Based Carriers

Delivery systems based on carbohydrates, including chitosan, cellulose polymers, starch-based materials, gum arabic and sodium alginate, have been widely used in pharmaceutical field to enhance absorption of bioactive compounds, owing to their biodegradability, biocompatible and non-toxic properties. Chitosan is the most widely used material as an EGCG carrier, for it is the most abundant alkaline natural polysaccharide with good biological intermiscibility and film-forming properties. Swiss Outbred mice oral administration of the chitosan-tripolyphosphate nanoparticles enhanced the plasma exposure of total EGCG by a factor of 1.5 relative to free EGCG [42]. In addition to chitosan, digestive stability of green tea catechins encapsulated into γ-cyclodextrin(γ-CD) or coated with hydroxypropylmethyl cellulose phthalate (HPMCP) was enhanced by 65.56% or 57.63%, respectively. Formulated catechins followed by encapsulation into γ-cyclodextrin significantly increased intestinal transport by 22.98% and 23.23% when coated with for S-HPMCP and L-HPMCP, respectively [59]. Bioavailability of the catechins increased by 4.08 and 11.71 times when coated with S-HPMCP and L-HPMCP, respectively, indicating that coating with HPMCP could be an effective way to improve the digestive stability and intestinal transport of catechins [60]. It is demonstrated that HPMCP will not dissolve in the acidic juices of the stomach (pH ≈ 3), but it will dissolve in the alkaline (pH ≈ 7–9) conditions of the small intestine. There was study showing that intestinal absorption of the enteric-coated ingredient by HPMCP increased due to its controlling both the erosion rate and the length of intestinal segment [59]. Overall, many carbohydrates can stabilize formulations and provide protection and sustained release of the EGCG.

#### 3.1.3. Lipid-Based Carriers

Lipid carriers incorporating drugs have been found to improve the absorption and circulation time in the body versus stand-alone compounds [61] and it is also used as nanodelivery systems to encapsulate green tea extract. Tea polyphenol nanoliposomes improved the stability of tea polyphenol in alkaline solution, resulting in better sustained release property, with equivalent antioxidant activities [62]. EGCG encapsulated in liposomes increased drug deposition by 20-fold as compared to the free form [63]. The same results have been confirmed by various tests. When the catechins encapsulated on liposomes were orally administered to rats, the catechinlevel in blood was enhanced at the later stage compared with the aqueous solution control. Additionally, elastic liposomes showed 2.9- and 2.7-fold higher catechin accumulation in the cerebral cortex and hippocampus, respectively [64]. The degenerations of in vitro antioxidant activities of EGCG were effectively slowed by nanoliposome encapsulation [39]. Test on niosomes, a kind of novel vesicular system with a bilayer containing nonionic surfactants and cholesterol, demonstrated that drug-loaded niosomes had stronger stability and lower toxicity than control, resulting in a significantly improved absorption compared to their free forms [36].

#### 3.1.4. Mechanism by Which Nano-Carriers Improving the Bioavailability of Catechins

Catechins are unstable and highly susceptible to ambient conditions like oxygen, pH changes, metal ions and other stress factors. Nanoparticle-based delivery systems are believed to be plausible options to protect catechins against harsh conditions. The EGCG loaded on lipids showed better stability. Many food formulations are water based, the presence of EGCG on liposomes had the potential to promote the formation of hydrogen bonds between hydrophilic catechins and polar head groups [65]. In vitro study shows that chitosan-tripolyphosphate (TPP) nanoparticles improved the stability of EGCG in alkaline solution [66]. EGCG encapsulated in chitosan exhibited higher stability than free EGCG in simulated gastrointestinal (GI) tract. It is hypothesized that the interaction between positively charged chitosan and negatively charged tea catechins formed polyelectrolyte matrices with stable spherical shape and pH-tunable dimensions, which overcame the poor gastrointestinal stability of tea catechins [67]. Moreover, transendothelial electrical resistance (TEER) experiments showed that the nanoparticles with a positive surface charge transiently opened the reversible tight junctions between Caco-2 cells and thus promoted the paracellular transport of tea catechins [68]. However, it is considered that the mechanism by which absorption of catechins is enhanced is likely due to the improved stabilization of catechins after encapsulation, but not through the effects of chitosan nanoparticles on intestinal paracellular or passive transcellular transport processes or on efflux proteins [69].

Although the half-life period of EGCG (5.0–5.5 h) is about two times longer than that of EGC or EC (2.5–3.4 h), it is still too short to exert clinical effect. Sustainable and control release of catechins is a feasible way to improve their bioavailability. However, sustained release capacities of EGCG depend on the carrier systems. About 6.0% and 12.6% EGCG was released from EGCG nanoliposomes at 6 and 24 h, respectively [39]. When EGCG was loaded on niosomes, the residual EGCG increased from 3% to 49% after incubation in simulated intestinal fluid for 2 h, meanwhile the niosomes loaded EGCG exhibited stronger antioxidant ability than free EGCG during intestinal digestion [70]. Chitosan coatings could control the release pattern of EGCG from the BSA-EGCG nanoparticles, with slow release of EGCG in the simulated acidic gastric juice conditions but faster release in simulated alkaline intestinal fluid [46,71].

Some nanoparticles not only enhance the stability of EGCG, but also improve the membrane permeation. Song [36] reported that the transport of drug-loaded niosomes may be contributed to the endocytotic pathway. The interaction of EGCG with the phosphate moiety of dimyristoyl-phosphatidylcholine (MDPC) lipid [72] which is one of the components of the cell membrane makes it easier to cross the membrane. Thus, entrapping EGCG into liposomes could protect EGCG from enzymatic degradation and improve the membrane permeation. Accordingly, liposomes are considered to be a good carrier to improve EGCG adsorption via oral ingestion.

In addition, the drug loaded on nanoparticles can protect the core material from adverse environmental conditions, delay its degradation in the humoral environment, make it easily penetrate various barriers to reach the target organs, which contributes to the controlled liberation of the drugs and improvement of the drug bioavailability. The effects of EGCG nanoparticles are listed in Table 1.

### 3.2. Molecular Modification

**Figure 2:**
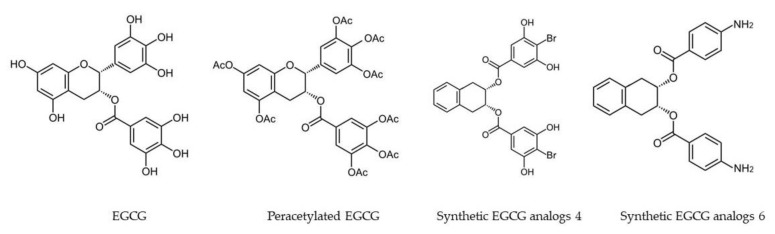
Molecular structures of (−)-epigallocatechin-3-gallate (EGCG) and EGCG-based prodrugs and analogs.

Biochemically, conjugation of the free hydroxyl groups surrounding the molecules influences the efficacy of ingested tea catechins. A molecular modification method using peracetate acid can be used to protect the reactive hydroxyl groups of EGCG. Synthesized peracetate-protected EGCG (AcEGCG, Figure 2) was found to be much more stable than free EGCG under slightly alkaline physiological conditions and showed greater efficacy in proteasome inhibition and induction of cancer cell death [87].

It was reported that AcEGCG increased EGCG stability, resulting in increased bioavailability and enhanced inhibition on the proteasome and growth of breast tumor. Peracetylation also increased the bioavailability of EGCG in esophageal and colon cancer cells [88], and the AcEGCG showed much higher potency to prevent dextran sulfate sodium (DSS)-induced colitis than free EGCG [6,89]. These studies suggest that AcEGCG has improved biological activity in vivo. This effect is accomplished by making hydroxyl groups unavailable for biotransformation or oxidative degradation. AcEGCG increased the intracellular levels of EGCG as a result of increased cell uptake, and convert intracellularly into EGCG in the presence of esterase, which could be detected within 5 min following incubation of AcEGCG in mouse plasma at 37 °C. However, once EGCG was released from AcEGCG, it is then subject to biotransformation and efflux at a rate similar to free EGCG [88].

Acetate protection of EGCG’s phenol groups is a useful tool for enhancing the stability and improving the bioavailability of EGCG. Furthermore, other chemical modifications that can improve the physiochemical properties of EGCG or reduce its biotransformation may be also useful in improving its bioavailability (Table 2).

### 3.3. Co-Administration of Catechins with Other Bioactive Components

It was found that a mixture of tea catechins and ascorbic acid significantly increased tea catechins’ recovery in a simulated in vitro digestion system [96]. Absorption of EGC and EGCG was significantly enhanced in formulations containing ascorbic acid and carbohydrate derives. Also, accumulation of EGC, EGCG and ECG by Caco-2 cells was significantly increased in formulations containing ascorbic acid and sucrose [59,97,98].

Apart from ascorbic acid, many other bioactive compounds show synergistic interactions with tea catechins. A major challenge for cancer chemotherapy and radiotherapy is time-dependence multi-drug resistance (MDR), as well as treatment interruptions caused owing to various side effects. To overcome this shortcoming, developing novel combination therapies for chemotherapy and bioactive dietary compounds will be a trend in future. The combination of catechins with other drugs which shows synergistic effects might be a promising approach.

Tea catechins showed a good coordination with some conventional anticancer drugs. The presence of EGCG significantly increased the pharmacokinetics of orally administered tamoxifen, which was attributed to the decrease in first-pass metabolism in the intestine and liver by inhibiting P-glycoprotein and CYP3A by EGCG [99]. Kitagawa et al. [100] reported that EGCG inhibited the efflux of P-gp substrates, verapamil and quercetin in KB-C2 cells, since the effects of EGCG were stronger than those of verapamil and quercetin, leading to the elevation of substrate accumulation. NS-398, a selective cyclooxygenase-2 inhibitors, showed an increased efficacy for inhibiting cancer cells when it was used jointly with EGCG by promoting apoptosis induction, inhibiting nuclear factor-Κb [101]. The combination of EGCG with sulindac significantly up-regulated expression of growth arrest related genes, with DNA damage-inducible 153 (GADD153) increasing by about 12-fold and p21WAF1 increasing by 3-fold; meanwhile no effects were observed when EGCG or sulindac was used alone [102] (Table 3).

## 4. Conclusions and Future Expectations

Low bioavailability of green tea catechins is an important factor leading to the observed inconsistency between in vitro and in vivo studies. Stability, absorption rate and efflux influence the bioavailability. Extreme pH conditions in the stomach and intestinal tract as well as related digestive enzymes are confirmed to be factors inducing instability including degradation and conjugation of catechins. Catechins are considered to be transported across intestinal epithelium principally by passive diffusion, including paracellular and transcellular diffusions because no specific receptors on the surface of small intestinal epithelial cells have been found to carry EGCG into cells, which is the main reason why the absorption rate of catechins is low. The active efflux of partial absorbed catechins decreases the plasma catechins concentration [43].

The bioavailability of catechins can be improved by nanostructure-based drug delivery systems, molecular modification and co-administration with some other bioactive ingredients. Encapsulation of tea catechins on protein-based, carbohydrate-based and lipid-based nanoparticles improved stability, sustainable release and cell membrane permeation of catechins, resulting in increased bioavailability. Molecular modification such as synthesizing peracetylated EGCG (AcEGCG) protects hydroxyl groups on EGCG from oxidative degradation until it is deacetylated into its parent EGCG by esterases in cells [87], which decreases biotransformation and efflux of EGCG. Co-administrating or formulating catechins with appropriate other drugs or bioactives will produce a synergism effects through interaction of catechins with the selected drugs, resulting in improvement of absorption and inhibition of efflux transporter [35,78,99,100].

There are still great expectations in the fields of bioavailability research of tea catechins. Disease-targeting and multiple molecular targeting are expected to be improved so as to find more potent, stable and specific active formulation of catechins [102]. Targeted delivery with rapid release of a toxic dose level is expected in cancer treatment. However, sustainable release of low dosage EGCG over a longer period of time is needed for treatment of chronic diseases like atherosclerosis or neurodegeneration. Furthermore, improving skin permeability is desired in the cosmetic field. More importantly, the effectiveness of these improved techniques requires further clinical validation.

## Figures and Tables

**Table 1:** Various carriers of catechins and their improvement of effectiveness.

Materials of Carrier	Bioactive	Improvement of Effectiveness	Ref.
Chitosan and TPP	EGCG	Improved stability and increased plasma concentrations of EGCG	[42,66]
Chitosan	Catechin and EGCG	Enhanced the intestinal absorption of catechins	[69]
Carboxymethyl chitosan	EGCG	Improved stability and sustained release.	[73]
Chitosan and γ-glutamic acid	Catechins	Increased the paracellular transport of catechins with effective antioxidant activity.	[68]
Chitosan and polyaspartic acid	EGCG	Improved the effectiveness of EGCG against rabbit atherosclerosis.	[74]
Chitosan and caseinophosphopeptides	EGCG	Enhanced the intestinal permeability of catechins	[75]
Beta-chitosan	Catechins	Improved the antibacterial activity	[76]
Chitosan or poly-ε-lysine	EGCG	Improved the stability of EGCG and improved the permeability across intestine	[47]
Chitosan	TP	Improved the level of radioprotection of TP.	[48]
HPMCP or γ-CD	Catechins	Increased intestinal transport.	[59]
HPMCP	Catechins	Improved the stability of catechins and increased intestinal transport.	[60]
Lipsomes	Catechins	Enhanced the transdermal delivery of catechins.	[77]
Lipsomes	Green tea extract	Improved the stability of catechins.	[38]
Lipsomes	TP	Improved the stability of catechins.	[62]
Liposome	Catechins	Inducted greater basal cell carcinomas death at lower concentrations.	[63]
Nanolipsomes	EGCG	Induced apoptosis and inhibited proliferation of MCF7 breast cancer cells.	[78]
Nanolipsomes	Catechins	Improved the antioxidant activity	[79]
Nanostructured lipid	EGCG	Inhibitd atherosclerotic lesion development through decreasing macrophage cholesterol content and monocyte chemoattractant protein-1 expression.	[80]
Nanolipidic	EGCG	Improved α-secretase inducing ability of EGCG for the treatment of Alzheimer’s disease.	[81]
Nanoethosomes	EGCG	Enhancing the skin permeability.	[82]
Niosomes	EGCG	Improved the stability of catechins and exhibited stronger antioxidant ability.	[70]
Ovalbumin	EGCG	Enhanced the apparent permeability coefficient of EGCG on Caco-2 monolayers	[46]
Casein micelles	EGCG	Improved the stability of catechins, and decreased the proliferation of HT-29 cancer cells without affecting the bioefficacy of EGCG.	[83]
Casein micelles	Catechins	Improved the stability of catechins, and decreased the proliferation of HT-29 cancer cells in a manner similar to that of free EGCG.	[53]
Nanoemulsion gel	Catechins	Showed sustained release profile and enhanced photoprotection potential due to its improved skin permeability and bioavailability through transdermal route.	[49]
Zein	EGCG	Improved the stability of EGCG.	[41]
Rice bran protein isolate	Catechins	Improved the stability of catechins.	[56]
β-lactoglobulin	EGCG	Protected antioxidant activity of EGCG	[84]
Selenium nanoparticles and Tet-1 peptide	EGCG	Inhibited amyloid-β fibrillation and disaggregate preformed amyloid-β fibrils into nontoxic aggregates.	[85]
poly(lactide-co-glycolide)	EGCG	Showed a superior ability to prevent DMBA-induced DNA damage at much lower concentrations	[86]

TPP: tripolyphosphate; BSA: bovine serum albumin; HPMCP: hydroxypropyl methyl cellulose phthalate; γ-CD: γ-cyclodextrin; DMBA: 7,12-dimethylbenzanthracene.

**Table 2:** Molecular modification of EGCG and its effects.

Molecular Modification	Tested Cell Lines	Cancer Type	Major Effects	Ref.
Peracetylated EGCG	Jurkat T	Leukemic	Being more stable than free EGCG at neutral pH and showing greater efficacy in proteasome inhibition and cell death induction.	[87]
	KYSE150, HCT116	Esophageal and colon	Increasing the biological potency in vitro and the bioavailability of EGCG in esophageal or colon cancer cells.	[88]
		Colon	Showing stronger prevention potency to DSS-induced colitis than free EGCG.	[89]
	CD34+	Skin	Preventing skin carcinogenesis by suppressing the PKD1-dependent signaling pathway in CD34+ skin stem cells and skin tumors	[90]
	MDA-MB-231	Breast	Increasing the bioavailability, stability, and proteasome inhibition and anticancer activities of EGCG in human breast cancer cells and tumors.	[91]
	CWR22R	Prostate	Being more stable, increasing the therapeutic anticancer effects in androgen-independent prostate cancer	[92]
		Endometrium	Inhibiting the growth, development and angiogenesis of experimental endometriosis in mice, with improved efficacy, bioavailability, anti-oxidation and anti-angiogenesis capacities.	[93]
			Inhibiting tumor angiogenesis through downregulation of VEGFA and HIF1α in tumor cell and chemokine(C-X-C motif) ligand 12 in host stroma.	[94]
Synthetic EGCG analogs 4 and 6 (Figure 2)	MDA-MB-231	Breast	Activating AMPK, with inhibition of cell proliferation, up-regulation of the cyclin-dependent kinase inhibitor p21, down-regulation of mTOR pathway, and suppression of stem cell population in human breast cancer cells.	[95]

**Table 3:** Improvement of biological activities by co-administration catechins with other complementary bioactives.

Tea Catechins	Complementary Bioactives	Effectiveness	Ref.
Catechins	Ascorbic acid (and sucrose or xylitol)	Increasing tea catechins recovery in a simulated in vitro digestion.	[96]
		Improving catechins bioavailability by enhancing bioaccessibility and intestinal uptake.	[97]
		Promoting intestinal transport of catechins in a dose-dependent manner.	[98]
		Increasing bioavailability of green tea catechins.	[59]
EGCG	Piperine	Increasing EGCG bioavailability by inhibiting glucuronidation and gastrointestinal transit.	[103]
	Rutin	Improving the stability and the prolonged release of rutin in simulated GI fluid, owing to the external attachment of EGCG to the ferritin cage, potentially reducing enzymolysis in GI fluid.	[104]
	Tamoxifen	Significantly improving the pharmacokinetics of orally administered tamoxifen.	[99]
		Promoting the suppressive effects on growth of ER-negative breast cancer, along with a decrease in expression of tumor proteins mTOR and the EGFR.	[105]
	Erlotinib	Inhibiting pEGFR and pAKT, increasing activation of caspases 9, 3 and PARP, inhibiting cell proliferation and inducing apoptosis.	[106]
		Inhibiting cancer cell proliferation, increasingresponse to erlotinib.	[107]
	Nicardipine	Increasing bioavailability of oral administered EGCG, resulting in inhibition both the hepatic CYP3A subfamily and intestinal P-gp.	[108]
	Oxcarbazepine	Enhancing the degree of systemic exposure tooxcarbazepine and licarbazepine in rats.	[109]
	Verapamil	Increasing significantly the bioavailability of verapamil.	[110]
	Caffeine	Enhancing the absorption of EGCG in humans.	[111]
	Genistein	Enhancing EGCG bioavailability and inhibiting tumorigenesis in mice.	[112]
	NS398	Enhanced apoptosis induction in vitro and tumor growth inhibition in vivo.	[101]
	sulindac	Inducing apoptosis of cancer cells by promoting the expression of GADD153 and p21WAF1 genes.	[102]
	Curcumin	Enhancing cell cycle arrest at G1and S/G2 phases.	[113]
		Synergistic cytotoxicity to the cancer cells along with G2/M-phase cell cycle arrest.	[114]
ECG and EGCG	Doxorubicin	Enhancing sensitivity of cancer cells to doxorubicin and the accumulation of doxorubicin in cancer cells.	[115]
Green tea polyphenol	Acetaminophen	Green tea polyphenol supplementation attenuated hepatotoxicity by normalizing cyclooxygenase andB-cell lymphoma-2 activation, suggesting a potential use for in treating acetaminophen toxicity.	[116]

Tamoxifen: (Z)-1-(p-Dimethylaminoethoxyphenyl)-1,2-diphenyl-1-butene; Erlotinib: *N*-(3-ethynylphenyl)-6,7-bis(2-methoxyethoxy)-4-quinazolinamine; Nicardipine: 5-*O*-[2-[benzyl(methyl)amino]ethyl] 3-*O*-methyl 2,6-dimethyl-4-(3-nitrophenyl)-1,4-dihydropyridine-3,5-dicarboxylate; Oxcarbazepine: 10,11-Dihydro-10-oxo-5h-dibenz[b,f]azepine-5-carboxamide; Verapamil: 5-[*N*-(3,4-Dimethoxyphenylethyl)methylamino]-2-(3,4-dimethoxyphenyl)-2-isopropyl-valeronitrile; Sulindac: (Z)-5-Fluoro-2-methyl-1-[p-(methylsulfinyl)benzylidene]indene-3-acetic acid.
